# Influence of digital health literacy on online health-related behaviors influenced by internet advertising

**DOI:** 10.1186/s12889-024-19506-6

**Published:** 2024-07-20

**Authors:** Giulia de Oliveira Collet, Fernanda de Morais Ferreira, Daniela Fernandes Ceron, Marina de Lourdes Calvo Fracasso, Gabriela Cristina Santin

**Affiliations:** 1 Department of Dentistry, State University of Maringá, Avenida Mandacarú, 1550, Maringá, PR 87080-000, Brazil; 2 Department of Child and Adolescent Oral Health, Federal University of Minas Gerais, Avenida Presidente Antônio Carlos, 6627, Belo Horizonte, MG 31270-901 Brazil

**Keywords:** Social Media, Health literacy, eHealth Strategies

## Abstract

**Background:**

The frequency of health-related information seeking on the internet and social media platforms has increased remarkably. Thus, the ability to understand and select accurate health-related information online, known as EHealth literacy, is crucial for the population. Therefore, this study aimed to evaluate how eHealth literacy influences the critical analysis of oral health-related information obtained from the Internet and its influence on oral health-related behaviors.

**Methods:**

A cross-sectional online study was conducted with 418 Brazilian adults who responded to the eHEALS questionnaire, with questions regarding the influence of online information on oral health decisions. Socioeconomic and demographic characteristics were also collected.

**Results:**

The mean eHEALS score was 27.85 (± 8.13), with a range spanning from 8 to 40 points. Participants with higher eHEALS scores reported using social media platforms to seek for dentists and to acque information about symptoms, diagnoses, and treatments. Furthermore, this group tended not to disregard professional health recommendations based on Internet information and abstained from utilizing products promoted by digital influencers. In multivariate models, increased eHEALS scores were associated with reduced consumption of products endorsed by digital influencers.

**Conclusions:**

The findings from this study suggest that individuals with higher scores on the eHealth literacy questionnaire often conduct research on their health status and seek for health care providers on the Internet. Moreover, these individuals were less likely to be influenced by digital influencers.

**Supplementary Information:**

The online version contains supplementary material available at 10.1186/s12889-024-19506-6.

## Introduction

Health-related information is widely available in the digital world, allowing Internet users to learn about symptoms, prevention, and treatment options for various diseases with just a few clicks [11, 25]. The impact of social media on health behaviors is becoming increasingly remarkable [25]. Social media has become so pivotal in the health sector that findings from previous research already demonstrated that a significant amount of patients now rely on these platforms to select doctors, dentists, and hospitals [27, 1, 4]. Thus social media has emerged as a significant resource for promoting health and positive lifestyle behaviors, such as physical activity, healthy diets, and seeking for social and emotional support [9, 8].

Importantly, a substantial portion of health information on social media is disseminated by digital influencers and users [22, 5] who establish trustworthy relationships with their followers. This dynamic can potentially promote adverse health risk behaviors such as increased alcohol consumption [22] and body image dissatisfaction [23]. Factors such as the individuals’ inability to verify the accuracy of the information contribute to the spread of health misinformation on the internet [12]. Beyond the large number of users, online social networks offer several advantages, such as high engagement, interactivity, and considerable influence over users [13].

In this context, for internet use in self-managing health to promote wellness rather than exacerbate health disparities, individuals must be able to seek health-related information online and, furthermore, to evaluate the quality of the information found to use it for a better decision making in health. This concept, known as digital health literacy, can be assessed by some validated instruments such as the eHealth Literacy Scale (eHEALS), developed by Norman and Skinner [21]. This tool relies on respondents’ self-analysis of their health-related digital skills. Higher eHealth literacy levels are linked to enhanced self-management in health [7, 18, 29], which is positively associated with better health-promoting behaviors and attitudes [30], to the best of our knowledge, this association has not yet been investigated in the context of oral health. Therefore, this study aimed to evaluate whether Brazilian adults with a higher level of digital health literacy are less susceptible to the influence of Internet on their oral health-related behaviors.

## Methods

This study was approved by the Human Research Ethics Committee of the State University of Maringá (certificate number: 39618720.0.0000.0104). A cross-sectional study with Brazilian adults was conducted with a non-probabilistic sample using the “snowball” method. The sample size was calculated to detect the difference between two means in independent groups, G1: Individuals with minor scores in digital health literacy and G2: Individuals with high scores in digital health literacy (www.calculoamostral.bauru.usp.br). Considering the results of a previous study [31], the estimated standard deviation of the eHEALS score was 0.61 and the minimum difference to be detected was 0.12 [31]. Thus, the minimum sample size for the study was 407 volunteers.

Data were collected between January and February 2021 using an online questionnaire designed through the Google Forms platform (Chart 1). The questionnaire was written in Portuguese and sent to participants through the message applications WhatsApp, Facebook, and Instagram. The initial WhatsApp groups included post-graduate students and patients who attended the University’s Dental Clinic, which made the sample more heterogeneous. The participants were free to send the questionnaire to other volunteers. The inclusion criteria were: age above 15 years and the agreement to participate in the data collection, given previously through an informed consent form.

The questionnaire comprised three steps (full information about the questionnaire items are available in Chart 1). The first section included the eHealth Literacy Scale (eHEALS) [21, 3], consisting of eight items with response options on a five-point scale: strongly disagree (1 point), partially disagree (2 points), undecided (3 points), partially agree (4 points), and strongly agree (5 points). The original formula proposed by Norman and Skinner [21] for calculating the score on the questionnaire is the arithmetic mean of the scores of each item,thus, the final score ranges from one to five points. In the present study, the final score was calculated by the sum of each item score and could, therefore, range from 8 to 40 points, with higher scores representing a better/higher level of digital health literacy. This form of determining the final score facilitates the interpretation of the questionnaire and was used in studies by Gazibara et al. [7] and Nguyen et al. [19].

The second section of the questionnaire included items on how respondents seek information related to health and oral health on the Internet and how they behave in regards of such information. The first three items of this section address the frequency with which respondents conduct online searches about dentists, symptoms, diagnoses, and treatments for oral and general health problems. The response options were arranged on a four-point scale: never, rarely, often and always.

The following questions were dichotomous (yes or no) and covered topics such as self-medication, the use of general health products endorsed by influencers (if yes, specifying the products used based on the recommendations of digital influencers), whether respondents had ever disregarded the recommendations of dentists due to information found online.

The third and last section comprised a sociodemographic and economic questionnaire with items on age, sex (male, female or “prefer not to say”), level of schooling (incomplete primary school, complete primary school, incomplete high school, complete high school, incomplete higher education, complete higher education and graduate studies), marital status (married, living with a partner, single, widowed or divorced), family income (using the Brazilian monthly minimum wage as reference), whether participants had easy access to the Internet and how it was accessed (mobile phone, computer/laptop, tablet or other), and whether they used social media. Lastly, respondents were asked if they were health care providers (physicians, dentists, nurses, etc.).

### Insert chart 1

For statistical purposes, age was divided into categories (‘ ≤ 34 years old’; ‘35 to 49 years old’, and ‘50 years old or older’). Regarding marital status, the “single”, “widowed”, and “divorced” categories were combined and the “married” and “lives with a partner” categories were also merged. Schooling was divided into equal to or more than 12 years of study (referring to higher education) and less than 12 years of study (for respondents who either completed or did not complete high school education). Income was divided into more than 5 times the monthly minimum wage (MMW) in Brazil and equals to or less than 5 MMW [15].

Data from the questionnaire was initially analyzed through descriptive statistics.. Statistical analysis was then performed with the IBM SPSS 22.0 software (SPSS Inc., Chicago, IL, USA), though association tests and binary logistic regression models, considering a 5% significance level. The Kolmogorov–Smirnov test was used to determine data distribution. The Mann–Whitney, Kruskal–Wallis, and chi-square tests for independent samples were used.

A multivariate binary logistic model was performed with the equation that best explains the dependent variable “use of health products recommended by influencers” and the independent variables socioeconomic and “eHEALS literacy score” which reached statistical significance in the bivariate analysis along with the confounding parameters (age, schooling and income).

The psychometric properties of the instrument were not evaluated in the study sample, as this was not the study’s objective and the instrument had been previously validated in a similar sample [31].

## Results

A total of 418 individuals answered the questionnaire. The female and male sexes accounted for 66.7% and 33.3% of the sample, respectively. Mean age was 35.6 ± 13.0 years old, and 70.3% of the sample had equal to or more than 12 years of schooling. The majority (56.9%) reported being single, divorced, or widowed, whereas 43.1% were married or lived with a partner. Sixty-one percent of the respondents had an income higher than five times the monthly minimum wage. Slightly more than a quarter of the participants worked in the health field (Table 1).

**Table 1:** Frequency of demographic-socioeconomic variables, eHEALS score, and use of social media among participants (*n* = 418)

*Variable*	*Category*	*n (%)*
Age	≤ 34 years	241 (57.6)
	35 to 49 years	106 (25.4)
	≥ 50 years	71 (17.0)
Sex	Female	279 (66.7)
	Male	139 (33.3)
Schooling	< 12 years	124 (29.7)
	≥ 12 years	294 (70.3)
Marital status	Married/lives with partner	180 (43.1)
	Single/widowed/divorced	238 (56.9)
Income	≤ 5 times monthly min. wage	163 (39.0)
	> 5 times monthly min. wage	255 (61.0)
Easy access to the Internet	Yes	416 (99.5)
	No	2 (0.5)
Device for accessing the Internet	Mobile phone	356 (85.2)
	Notebook/Tablet	62 (14.8)
Use social media	Yes	403 (96.4)
	No	15 (3.6)
Works in health field	Yes	112 (26.8)
	No	306 (73.2)
Consulted the dentist´s social media	Rarely/Never	226 (54.1)
	Often/Always	192 (46.0)
Searched for information on the diagnosis or treatment proposed by dentist	Rarely/Never	243 (58.0)
	Often/Always	175 (42.0)
Looked up information on their symptoms before seeking health care provider	Rarely/Never	122 (29.2)
	Often/Always	296 (70.8)
Self-medications	Yes	162 (38.8)
	No	256 (61.2)
Used health product recommendations from influencers	Yes	92 (22.0)
	No	326 (78.0)
Ignored the counseling of the dentist	Yes	23 (5.5)
	No	395 (94.5)
		*Mean (*± *DP)*
eHEALS Score (continuous variable)	27.8 (± 8.13)	

Only two (0.5%) participants reported not having easy access to the Internet, while 356 (85.2%) indicated that they access the Internet primarily through a mobile phone. Fifteen participants (3.6%) reported not using social media platforms (Table 1).

### Searching for social media profiles about health and oral health on the internet

Most participants (70.8%) checked information on their general or oral health state before seeking a health care provider; 42.0% searched for information on the diagnosis or treatment proposed by a dentist, while 46.0% consulted the dentist’s social media platforms prior to scheduling an appointment. A total of 38.8% reported self-medicating based on information available on the Internet, and only 5.5% reported having ignored the advice of a oral health care provider due to information found online (Table 1).

Approximately 22.0% of the participants had used health products endorsed by digital influencers, the most cited of which were dietary supplements, beauty products, medications, and tooth whiteners, such as toothpaste with activated charcoal and bicarbonate.

**Fig. 1:**
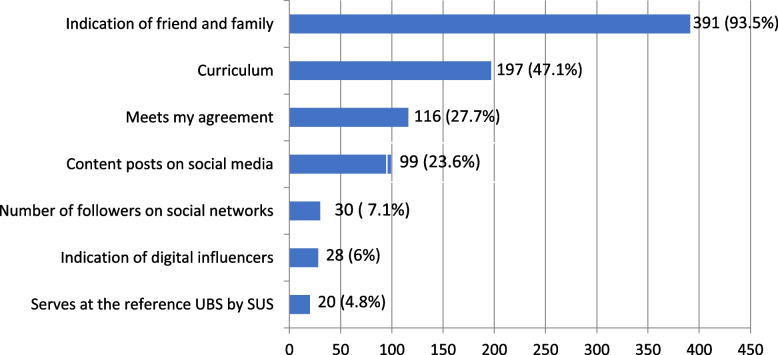
Reasons for choosing a dentist

Regarding factors influencing the choice for a dentist, 23.6% considered the publication of content on social media platforms significant, whilst 7.1% considered the number of followers to be important. However, recommendations from friends and relatives held the greatest influence in choosing a dentist (Fig. 1).

### EHEALS questionnaire

The mean eHEALS score was 27.8 ± 8.13 with a median score of 30 points. No statistically significant differences were found between the eHEALS literacy score and sex, age, marital status, or the use of social media (Table 2). Level of schooling, income, and being a health care provider were the only socioeconomic variables for which the eHEALS scores statistically differed according to the categories. Health care providers as well as individuals with more schooling and income demonstrated higher eHEALS scores (Table 2).

**Table 2:** Difference between the score on eHEALS questionnaire according to socioeconomic variables (*n* = 418)

		Score on eHEALS Questionnaire					
Socioeconomic variables	Category	Mean	Median	Min	Max	SD	*p*-value*
Sex	Female	28.2	30.0	8	40	8.00	0.208
	Male	27.1	29.0	9	40	8.37	
Schooling	< 12 years	24.6	26.0	8	40	9.50	< 0.001*
	≥ 12 years	29.2	31.0	9	40	7.06	
Marital status	Married/lives with partner	27.0	29.0	8	40	8.90	0.173
	Single/widowed/divorced	28.5	30.0	9	40	7.34	
Works in health field	Yes	32.1	32.0	9	40	5.80	< 0.001
	No	26.2	28.0	8	400	8.29	
Age	≤ 34 years	28.4	30.0	9	40	7.45	0.294
	35 to 49 years	27.5	30.5	8	40	8.81	
	50 years or older	26.3	28.0	8	40	9.15	
Uses social media?	Yes	28.0	30.0	8	40	8.00	0.202
	No	25.0	26.5	11	38	10.17	
Income	≤ 5 MMW	26.7	28.0	8	40	8.81	0.002
	> 5 MMW	28.9	31.0	9	40	7.46	

*Max. *Maximum value*, Min. *Minimum value*, SD *Standard deviation*, MMW *Minimum wage

*Significant difference (*p* < 0.05); Kruskal–Wallis and Mann–Whitney tests

Individuals with higher eHEALS did not usually ignore the recommendations of physicians due to information found on the Internet and were less likely to use health products endorsed by digital influencers. Table 3 displays the differences between the eHEALS scores among the categories of the questions on the use and search for information online.

**Table 3:** Difference between the score on eHEALS questionnaire according to online search profile and behaviors (*n* = 418)

		Score on eHEALS questionnaire					
Online search profile	Category	Mean	Median	Min	Max	SD	*p*-value*
Searches for information about dentist on social media	Never	24.2	26.0	8	40	9.83	< 0.001*
	Rarely	27.1	30.0	10	40	8.25	
	Often	29.7	30.0	14	40	5.90	
	Always	30.5	31.5	9	40	6.50	
Searches for information about diagnosis or treatment	Never	23.1	23.5	8	40	9.65	< 0.001*
	Rarely	26.7	28.0	10	39	7.66	
	Often	31.2	31.0	10	40	5.90	
	Always	31.1	32.0	9	40	6.29	
Searches for information about symptoms	Never	18.5	19.0	8	30	8.20	< 0.001*
	Rarely	25.8	26.0	10	40	8.54	
	Often	27.9	30.0	8	40	7.55	
	Always	31.2	31.0	13	40	6.45	
Has self-medicated	Yes	27.8	30.0	8	40	7.94	0.680
	No	27.8	30.0	8	40	8.26	
Ignored counseling of health care provider due to information found online	Yes	22.3	21.0	10	35	8.43	0.002*
	No	28.1	30.0	8	40	8.01	
Used products due to endorsement by digital influencers	Yes	24.7	27.5	9	38	8.34	< 0.001*
	No	28.7	30.0	8	40	7.87	

*Max.* Maximum value, *Min.* Minimum value, *SD* Standard deviation

*Significant difference (*p* < 0.05)*; *Kruskal–Wallis and Mann–Whitney tests

The results of the binary Logistic regression model with the dependent variable “use of health products recommended by influencers”, demonstrated that the variables “age”, “schooling”, and “income” were not significant; however, the variable “eHEALS literacy score” maintained statistical significance in the model (Table 4).

**Table 4:** Multivariate model for the use of health products indicated byinfluencers (*n* = 418)

	Unadjusted comparison			Binary logistic regression model			
Variable	n (%) “no” or Mean (SD)			*p* *	OR	CI95%	
**Income**	NO	YES	p*				
≤ 5 MMW	119 (73.0)	44 (27.0)	0.050	0.591	0.868	0.517—1.457	
> 5 MMW	207 (81.2)	48 (18.8)					
**Schooling**							
< 12 years	86 (69.4)	38 (30.6)	0.006	0.191	0.694	0.401—1.200	
≥ 12 years	240 (81.6)	54 (18.4)					
**Age**							
≤ 34 years	184 (76.3)	57 (23.7)	0.185	0.226	1.00		
35 to 49 years	82 (77.4)	24 (22.6)		0.088	1.908	0.909—4.004	
50 years or older	60 (84.5)	11 (15.5)		0.158	1.800	0.796—4.068	
**eHEALS Score (Continuous variable)**	28.7(7.87)	24.7(8.34)	< 0.001	0.001*	0.948	0.920—0.977	

*Significant difference (*p* <0.05)

## Discussion

These findings show that individuals with higher scores on the eHEALS questionnaire frequently conducted more searches on the Internet about health. Such individuals are also more critical with regards to online content, as they usually do not disregard the advice of health care providers when faced with information found online and do not use health products based on the endorsement of digital influencers.

Significant associations were identified between the level of media health literacy and both income and schooling. Consistent with previous studies [18, 31, 3], the findings from this study indicate that individuals with higher income and schooling also demonstrate a higher level of eHealth literacy. In contrast, no significant associations were observed between the eHEALS scores and sex or age, which is in agreement with findings described in the study conducted by Yamaguchi and collaborators (2020) also involving a sample of Brazilians.

The search for health-related information on the Internet is frequent [29] and the findings from previous studies show that individuals with higher eHEALS scores are more likely to actively seek and put into practice in their daily lives such online content [18]. This includes setting positive lifestyle behaviors, such as a healthy diet and regular physical activity [26]. Similar results were found in the present study, as individuals with higher eHealth literacy scores were more likely to engage in online searches about the symptoms of certain diseases and the diagnoses or treatments proposed by a dentist.

No significant association was found between the practice of self-medication and the score on the eHEALS questionnaire. This suggests that individuals, even those who considered themselves unable to conduct online health searches or distinguish between safe and unsafe sources, reported to self-medicating based on information obtained online. The unauthorized use of medications without a prescription from a physician became a more common occurrence during the coronavirus pandemic [14, 16]. This practice poses risks, including the potential for overdose and adverse drug interactions. Moreover, self-diagnosis may lead to inaccuracies [17].

While the most frequently accessed websites for health-related information were not investigated in the present research, a previous study carried out in Hong Kong showed that laypersons predominantly rely on online encyclopedias such as Wikipedia, and “question and answer” websites as Yahoo! Answers. The study further indicated that only a minority of individuals confirmed the accuracy of such information with a health care provider [29]. Another study conducted in Kuwait observed that most individuals use YouTube to access information about their health [2]. While Wikipedia may be considered a valuable source for general health subjects, there is no evidence it is a 100% reliable source [24].

The search for the dentist profile on social media and the content posted emerged as an important factor in the selection of a health care provider. Thus, physicians, dentists, and other care providers should work together to offer high-quality health-related information, as these professionals exert considerable influence over laypersons [29]. Due to the high engagement, interactivity, and influence on users, online social networks have been used as a strategy for improving the impact of health campaigns and promoting health care providers [20].

One issue with social media lies in its potential to influence the public both positively and negatively. In the current investigation, 22.0% of respondents reported using health products recommended by digital influencers – famous people on the Internet without adequate qualifications to provide health guidance, particularly among those individuals with low eHealth literacy. Medications, supplements, and beauty products were among the most cited products, such as tooth whiteners with activated charcoal and bicarbonate, known to be harmful to oral health, do not provide a whitening effect, and are not recommended by dentists [6]. However, our research results indicate that people who did not use products endorsed by digital influencers showed higher eHealth literacy levels, regardless of age, schooling, and income. This finding may suggest a potential protective factor against such influences.

Having a high level of eHealth literacy proves beneficial when conducting online health searches, knowing when to seek professional assistance and managing signs and symptoms. On the other hand, it is not recommended for laypersons to make important health decisions solely based on online information. Indeed, false health-related information is deliberately disseminated on the Internet for commercial or ideological motives [28].

This study revealed that individuals with a higher level of media health literacy were more critical with regards to information available on the Internet. Specifically, they were more critical of recommendations from digital influencers, and were less likely to disregard the advice of dentists due to information encountered online. Kim and Oh [10] also showed that eHealth literacy improves search behavior for health-related information on the Internet, contributes to health-promoting attitudes towards the information found, and boosts motivation for self-care among individuals with regards to their health. Thus, eHealth literacy may be associated with more informed decisions and choices in health matters, assisting in the self-management of health conditions.

Recognizing the considerable search for health-related information on social media platforms and the significant influence of these networks on users, it is important for health care providers to offer easily accessible, high-quality information [29]. Moreover, public health agencies should use online media as an effective, low-cost means of publicizing health campaigns.

This study has limitations that should be considered. Firstly, the eHEALS questionnaire relies on the self-analysis of one’s digital media capacities, a process prone to potential errors. Secondly, the sample selection, due to the employed methodology, is not representative of the broader Brazilian population. Furthermore, cross-sectional studies do not allow establishing a cause-and-effect relationship or analyzing behavior over time. Given the importance of eHealth literacy for enhanced self-management of health conditions, further studies should be conducted to comprehensively evaluate eHealth literacy.

## Conclusions

The findings from this study show that income and schooling are associated with higher levels of eHealth literacy. Notably, individuals with higher scores on the questionnaire explore more frequently the social media profiles of dentists before scheduling appointments, make online surveys about their general and oral health status as well as the diagnoses or recommended procedures. Furthermore, such individuals tend not to disregard the advice of health providers when faced with information found online. Individuals with a high level of eHealth literacy are also more critical of information available on the Internet and are less influenced by digital influencers.

### Supplementary Information

Supplementary Material 1.

## Data Availability

The datasets used and/or analyzed during the current study are available from the corresponding author on reasonable request.

## Abbreviation

eHEALS: EHealth Literacy Scale
