# Supplementary material for: Influence of digital health literacy on online health-related behaviors influenced by internet advertising
Source: BMC Public Health. 2024 Jul 20;24:1949. doi: 10.1186/s12889-024-19506-6 (PMC11265096; doi:10.1186/s12889-024-19506-6)
Supplement: Supplementary file 1 — Supplementary Material 1. [file 12889_2024_19506_MOESM1_ESM.docx]

**Chart 1: Questionnaire used for data collection.**

| **Section 1 of 3 – Media literacy questionnaire (eHealth Literacy Scale)**  Mark the response that best fits each statement. | |
| --- | --- |
| **Statements** | **Response** |
| 1. I know what health resources are available on the internet. | a) Strongly disagree  b) Partially disagree  c) Undecided  d) Partially agree  e) Strongly agree |
| 1. I know where to find helpful health resources on the internet. |  |
| 1. I know how to find helpful health resources on the internet. |  |
| 1. I know how to use the internet to answer my questions about health. |  |
| 1. I know how to use the information about health that I find on the internet to help me. |  |
| 1. I have the skills to evaluate the health resources that I find on the internet. |  |
| 1. I know how to differentiate reliable content from low-quality content about health on the internet. |  |
| 1. I feel confident in using information from the internet to make health decisions. |  |

| **Section 2 of 3 – Profile of research about health on the internet** | |
| --- | --- |
| 1. Before seeing a dentist, do you seek information about him on social media? | a) Always  b) Often  d) Rarely  e) Never |
| 1. After an appointment with the dentist, how often do you seek information on the internet about the diagnosis or procedure suggested by the dentist? |  |
| 1. How often do you seek information about your health state (symptoms) before seeing a health care provider? |  |
| 1. Have you ever made use of self-medication based on information available on the internet? | a) Yes  b) No |
| 1. Have digital influencers ever led you to consume any health-related product? |  |
| 1. If so, what products have you used due to recommendations from influencers? | Open question |
| 1. Have you ever ignored the counseling of the dentist due to information found on the internet? | a) Yes  b) No |
| 1. What do you take into consideration when scheduling an appointment with a dentist (you can mark more than one option) | a) Number of followers on social media  b) Posts of content on social media (publications, live streaming, stories)  c) Résumé  d) Indication from friends or relatives  e) Indication from digital influencers (someone famous on the internet)  f) What he/she treats at the primary care unit of reference in the public healthcare system  g) Accepts my health insurance plan |

| **Section 3 of 3 – Socioeconomic and demographic questionnaire** |  |
| --- | --- |
| 1. How old are you? | Open question |
| 1. What is your sex? | a) Female  b) Male  c) Prefer not to say |
| 1. What is your level of schooling? | a) Incomplete primary school  b) Complete primary school  c) Incomplete high school  d) Complete high school  e) Incomplete higher education  f) Complete higher education  g) Postgraduate studies |
| 1. What is your marital status? | a) Married  b) Single  c) Live with partner  d) Divorced  e) Widowed |
| 1. Do you have easy access to the internet? | a) Yes  b) No |
| 1. What device is your main form of accessing the internet? | a) Mobile phone  b) Tablet  c) Computer/laptop  d) Other |
| 1. Do you use social media? | a) Yes  b) No |
| 1. Are you a health care provider? | a) Yes  b) No |
